# Making Sense of OA

**DOI:** 10.1093/ijnp/pyu108

**Published:** 2015-02-16

**Authors:** J. Markovac, D. Malicke

**Affiliations:** Learning Design and Publishing, Medical School Information Services, University of Michigan, Ann Arbor, MI.

January, 2015 – This issue of the *International Journal of Neuropsychopharmacology* (IJNP) is available Open Access (OA), representing a major shift for the official *Journal of the International College of Neuropsychopharmacology* (CINP). Moving the journal from a traditional subscription-based publication model to an (OA) model means anyone in the world with Internet access can read and make use of all the important findings presented in the journal, free of charge. This new publication model will also make the papers published in IJNP more visible and thus more frequently downloaded, as well as allow much more flexibility for the journal’s authors to use and repurpose their own works.

The term “OA” has at least three primary definitions. One of the originals, known as the Budapest OA Initiative, requires that OA content be freely available and that all uses, including commercial uses, be allowed so long as the authors are “properly acknowledged and cited.” Others, such as the Bethesda and Berlin statements, say that OA materials should allow others to “copy, use, distribute, transmit and display the work publicly and to make and distribute derivative works, in any digital medium for any responsible purpose, subject to proper attribution…”. A key phrase to take note of here is “responsible purpose.”

Some OA supporters feel that to be truly OA, commercial uses must be allowed, which is in accordance with the Budapest definition. Others believe that allowing all commercial uses may be irresponsible and so interpret the Bethesda and Berlin statements to mean that OA materials should be made freely available for all noncommercial purposes, such as for education and research. Additionally, articles that are made freely available but do not permit any reuses without direct permission from the copyright holder are often referred to as “publicly available” or “free to view.”

And so it follows that copyright is an important factor in OA publishing. Authors who publish in OA journals or choose to make their articles available openly via an OA option generally retain copyright to their work. This is in contrast to traditional subscription-based journals, which require that authors assign copyright either to the publisher or to the learned society owner of the journal. Because authors keep copyright, they must assign a specific license to their work that provides guidance for the reader and for the journal regarding reuse of the copyright-protected material. There are many different licenses currently in use, most created by third parties, including publishers, other industry organizations, and not-for-profits such as Creative Commons (CC). The CC licenses are the ones most frequently used by open OA journals. There are several types of CC licenses that range in level of reuse restriction (Table 1).

**Table 1.** Creative Commons Licenses

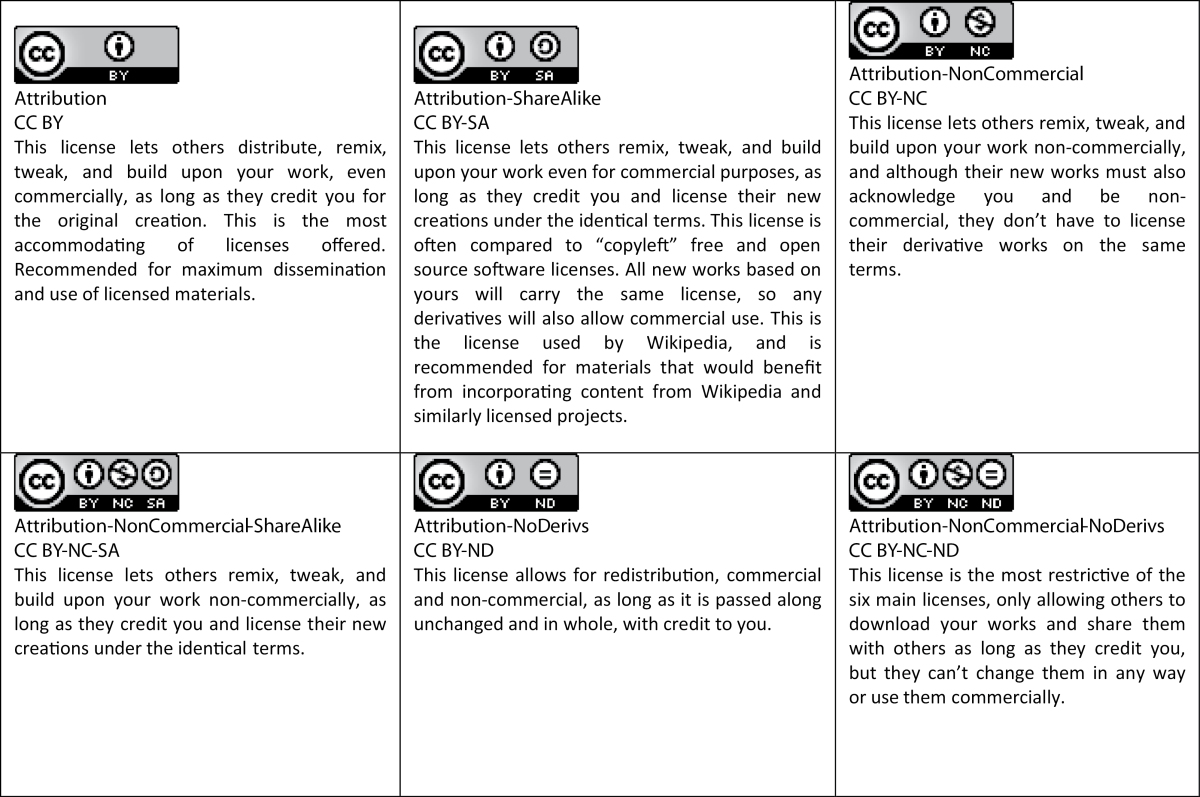

“Creative Commons Licenses” by Creative Commons is licensed under CC-BY 4.0

Most OA journals use either the CC BY or CC BY-NC license, some offering a choice to authors. Several funding agencies have mandated assignment of one of the most liberal licenses, CC BY, to articles resulting from research funded by those bodies. IJNP has adopted the CC BY-NC license, which is common for journals that publish materials with potential commercial value. In addition, this license prevents commercial exploitation of personal data collected in medical research and helps protect author reputation with respect to how the materials are reused. Authors are asked to assign this license when they submit their papers. An exception is made for those who are supported by RCUK/Wellcome Trust—these authors are asked to assign the CC BY license in order to be in compliance with their funders.

Initially intended for primary research journal articles, OA has since expanded to include other types of materials (eg, review articles, book chapters, teaching materials). The “modern” OA movement is thought to have started in the 1950s, but it did not become prominent until the 1990s when the Internet started to take hold and as a response to journal publishers’ high subscription prices. In life and biomedical sciences, OA gained momentum during the NIH leadership of Harold Varmus. As Director of the NIH, Varmus proposed a new “journal” to serve as a platform for preprints as well as peer-reviewed articles that would eventually become PubMedCentral (Varmus, 1999; NIH, 1999).

In 2000 Varmus, then at Memorial Sloan-Kettering Cancer Center, Pat Brown from Stanford, and Michael Eisen from Berkeley started a petition among the scientific community calling for a ban on publishing in any journal that did not make its content freely available online either upon publication or at most after a 6-month embargo. While tens of thousands of scientists signed the petition, most did not follow through, and in 2001 Eisen and Brown announced that they would start their own publishing company that would provide free online access to all articles. This publishing company, The Public Library of Science (PLOS), became one of the first major OA journal publisher when it launched in 2002 with the support of the Gordon and Betty Moore Foundation. Others soon followed suit.

**Figure 1.**
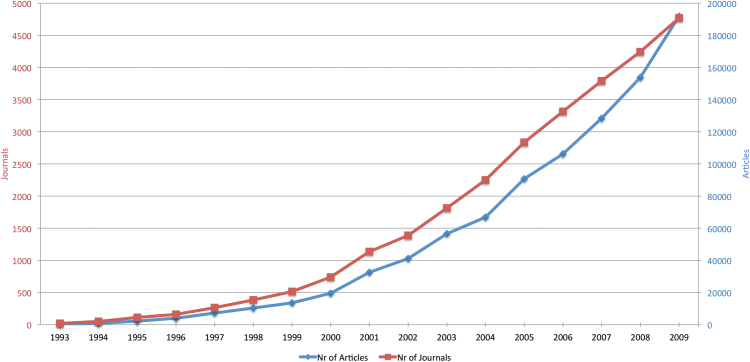
Development of OA (Laakso et al., 2011)

Figure 1 illustrates the increase in OA journals and articles from 1993 until 2009 (Laakso et al., 2011). Since then, the number of OA journals has increased even further, as has the number of articles. The OA journal PLOS One has published more than 105,000 papers since 2006. A recent article in *Nature* suggests that this kind of growth may be increasing to such an extent that it is becoming difficult for peer-review to keep up (Arns, 2014).

Some OA publishers are commercial companies, for example, BioMedCentral (a division of Springer), while others are not-for-profit (eg, PLOS). In navigating the world of OA publishing, one often comes across terminology that designates different types of OA: green and gold. Green OA refers to deposition of articles into a publicly accessible subject repository or an institutional repository. These can be preprints, authors’ final peer-reviewed manuscripts, or final journal articles. Since articles that are deposited into repositories as green OA and then published in a traditional subscription-based journal are generally under copyright and not licensed under any CC license, there are those who would argue that green OA is not true OA. In contrast, gold OA is OA publishing in a (peer-reviewed) journal. The journal can be a traditional subscription-based publication or fully OA. The former will offer an OA option to authors whereby, for a fee (known as an article processing charge or APC), an article can be made publicly available upon publication. The latter will generally charge APCs to authors, or the APCs may be paid by institutional subsidies.

**Figure 2.**
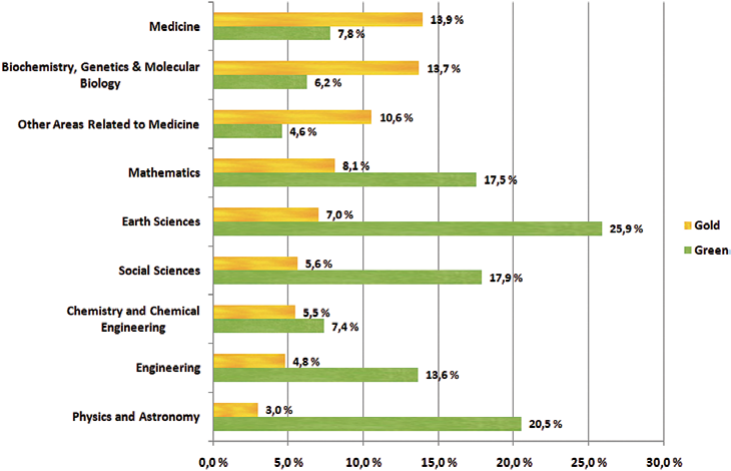
Green and gold OA by discipline (Bjork et al., 2010)

Figure 2 illustrates the relative distribution of OA articles by discipline (Bjork et al., 2010). The study found that chemistry had the lowest overall share of OA publications while earth sciences had the highest. In chemistry, biochemistry, and medicine, publication in OA journals (gold OA) was more common than self-archiving (green OA).

To date, there are more than 10000 gold OA journals in the Directory of OA Journals and many more that do not qualify for the directory due to questionable editorial and publishing practices. The latter are commonly known as “predatory journals” or “predatory publishers.” Jeffrey Beall, a librarian from University of Colorado Denver, has compiled a list, updated annually, of predatory journals and publishers, known as “Beall’s list” (Beall, 2014). While there have been cases of publishers claiming to be misrepresented as predatory, the list is a useful guide for potential authors, especially those who may receive email solicitations to submit papers to journals with titles remarkably similar to established, legitimate publications. Authors beware: there are many OA journals, but quality and reputation vary widely.

Whether or not a journal charges APCs varies widely among disciplines. OA journals in some fields, such as the arts and humanities, tend to have no fees, while others, especially in the “hard sciences”, charge APCs more often. Koziak and Hartley (2013) published a nice study of publication fees among OA journals across disciplines. Interestingly, that paper is not OA and could only be obtained with a subscription to the *Journal of the Association for Information Science and Technology* or by pay-per-view ($6 to rent for 48 hours, $15 to read, or $35 to read, print, and save)

Many reputable (and all predatory) OA journals in life and biomedical sciences and in medicine require authors to pay APCs, which can range from several hundred dollars to several thousand, depending on the journal and the discipline. A notable exception is e-Life (a joint venture between the Howard Hughes Medical Institute, the Wellcome Trust, and the Max Planck Society), an OA journal that launched in 2012, is fully supported, and at this time does not charge APCs.

For many journals, especially society publications, APCs are often similar to other publication fees (eg, page charges, submission charges). These standard publication fees are assessed to authors for subscription-based journals even where authors do not retain copyright and the articles are not publicly available or reusable on publication. Specifically for the IJNP, now that the journal is fully OA (gold OA), the APC for CINP members who are current on their dues payments is $1500 while the nonmember rate is $2200. Authors from developing countries are eligible for reduced APCs (see IJNP author instructions for details). There are no additional fees.

A great deal of research has been done on the impact of OA on citations, with mixed results. Several studies seem to show that OA does lead to more citations (Gargouri, et al, 2010; Jaing, et al, 2013) while others suggest that there is no statistical difference (Davis, 2008, 2011). Self-selection may be a factor as is the particular discipline or field of study (Davis, 2008). As OA publications continue to grow and expand, further research will be required to determine what the impacts are on various metrics and how significant they may be in the long run.

Many funding agencies and an increasing number of universities and countries have established mandates for grant recipients and university researchers to make their scientific articles available publicly, usually after a set embargo period postpublication. Furthermore, most of the funders’ regulations include mandatory deposit of the article to PMC or an equivalent repository or in university repositories. Specific requirements vary by institution, funding agency, and country. In some cases, the granting agency will provide funds especially designated to pay OA fees and will stipulate that the final journal article be deposited and made freely available upon publication without an embargo. Wellcome Trust is an example. HHMI, on the other hand, mandates that the final article be publicly available no more than 12 months after publication. Other grant agencies, such as the National Institutes of Health in the United States, require that the final accepted author manuscript (after all revisions but prior to copyediting and coding) be deposited to PMC and made publicly available following a 12-month embargo. In 2013, the Unites States Office of Science and Technology Policy directed all federal agencies with research and development budgets of more than USD100M to develop plans for making published results of federally funded research publicly available within 12 months postpublication. To date, all the US federal agencies have submitted their plans, but most of these have yet to be released.

A recent OA survey of researchers indicates that OA will continue to have a high profile and that the overall positive attitude toward this publishing model will grow. The study also showed that authors may not fully understand the implications of the various licenses and are unsure of the policies regarding repository deposits (Taylor and Francis, 2014). These results suggest there is an opportunity for publishers and learned societies to become more involved in educating authors about the many choices that are offered to them as they decide how to publish their work. With its recent decision to shift IJNP from a traditional subscription-based journal to a much more visible and widely distributed OA publication, the CINP has taken a significant step in promoting open scholarly communication and maximal dissemination of important research findings worldwide.
